# Perception of health risks in contexts of extreme climate change in semiarid Northeastern Brazil: an analysis of the role of socioeconomic variables

**DOI:** 10.1186/s13002-023-00597-1

**Published:** 2023-06-11

**Authors:** Valdir de Moura Brito Júnior, Henrique Fernandes de Magalhães, Ulysses Paulino Albuquerque

**Affiliations:** Laboratório de Ecologia e Evolução de Sistemas Socioecológicos (LEA), Departamento de Botânica, Universidade Federal de Pernambuco, Av. Prof. Moraes Rego, 1235, Cidade Universitária, Recife, Pernambuco 50670-901 Brazil

**Keywords:** Extreme climate events, Human health, Adaptive strategies, Climate vulnerability

## Abstract

**Background:**

Global climate change poses a significant challenge in contemporary society, particularly affecting vulnerable populations like small farmers residing in arid and semiarid regions. This study aims to investigate the perception of health risks and adaptive responses in the semiarid region of Northeast Brazil (NEB). Four questions were formulated: (1) How do socioeconomic factors influence the perception of health risks during extreme climate events? (2) How do socioeconomic factors impact the adoption of adaptive responses to mitigate health risks during extreme weather events? (3) How does the perceived risk level affect the utilization of adaptive responses? (4) What is the influence of extreme climate events on the perceived risks and the adoption of adaptive responses?

**Method:**

The research was conducted in the rural community of Carão, situated in the Agreste region of the State of Pernambuco, NEB. Semi-structured interviews were conducted with 49 volunteers aged 18 and above. The interviews aimed to gather socioeconomic information, including sex, age, income, access to healthcare services, family size, and education level. Additionally, the interviews explored the perceived risks and responses employed during different extreme climate events such as droughts or heavy rainfall. The perceived risks and adaptive responses data were quantified to address the research questions. Generalized linear models were employed to analyze the data for the first three questions, while the nonparametric Mann–Whitney test was used for the fourth question.

**Results:**

The study found no significant differences in the level of perceived risk and adaptive responses between the two climate extremes. However, the quantity of adaptive responses was found to be directly influenced by the perceived risks, regardless of the type of extreme climate event.

**Conclusion:**

The study concludes that risk perception is influenced by various complex factors, including socioeconomic variables, and plays a critical role in the adoption of adaptive responses during extreme climate events. The findings suggest that specific socioeconomic variables have a more pronounced influence on how individuals perceive and adapt to risks. Furthermore, the results indicate a cause-and-effect relationship between perceived risks and the generation of adaptive responses. These findings contribute to a better understanding of the factors shaping risk perception and provide valuable insights for future studies in regions prone to extreme climate events.

## Background

Climate change corresponds to changes in climate patterns on a global or regional scale over time [1]. The effects of these changes are felt differently depending on the region of the planet, with arid and semiarid regions being the most vulnerable to extreme climate events, such as droughts, floods and heatwaves [2]. Such adverse effects in semiarid regions make them potential risk areas for the health of human communities living in these environments [3, 4].

The latest assessment from the Intergovernmental Panel on Climate Change (IPCC) highlights the significant impact of human influence on our atmosphere, oceans, and land, resulting in noticeable warming. Furthermore, the report emphasizes that human activities have contributed to an increased occurrence of extreme climate events like droughts and heatwaves since the 1950s. Scientists assert that global warming will persist in the short term (2021–2040), primarily due to the continued rise of CO2 emissions in most projected scenarios. Should CO2 emissions maintain their current levels, which the IPCC considers as intermediate, it is likely that temperatures will increase by 1.5 °C [1]. These findings underscore the urgent need for concerted action to address climate change and its potential consequences. Such predictions indicate that semiarid regions may become even more vulnerable, as the worsening of the water deficit, added to the increase in temperature, may lead to an increase in the frequency of diseases, especially in low-income communities [2].

In a global scenario, Watts et al. [5] argued that small changes in the rainfall regime, or in temperature, can turn poor regions of underdeveloped countries into real centers of disease transmission through water and animal vectors. Therefore, climate change can lead to the emergence of new pandemics, a major challenge with health, environmental and economic implications [6–8]. Studies show that in the Brazilian semiarid region, drought, the most frequent climatic extreme in the region, is one of the main causes of infectious diseases, respiratory diseases and diseases transmitted by vectors [9, 10]. In addition to droughts, the Brazilian semiarid region is also vulnerable to floods and heavy rains, [11] which, in turn, are responsible for several diseases transmitted by bacteria, viruses, protozoa and worms [12].

With the increase in the frequency of extreme climate events, institutions around the world have turned to research on climate change, its consequences, and its effects in all fields of society [10, 13, 14]. Some of these studies have focused on understanding people's perception of climate change [15, 16]. Others, in turn, seek to understand how people perceive the risks caused by climate change and how they adapt to these risks [16–23].

For the purposes of this study, we understand risk as the exposure of an individual or group of individuals to potentially unfavorable circumstances [24]. In turn, risk perception can be defined as an individual’s set of judgments, feelings, attitudes and beliefs regarding risk assessment. [25]. Adaptation, in the context of climate changes, refers to individual or collective behavioral adjustments that aim to improve practices that reduce vulnerability to real or expected climate changes [26].

Among the aspects that can influence the perception of risk, as well as the adoption of adaptive responses, socioeconomic factors have been widely used in the scientific literature in different areas, including public health [27]. With the potential increase in risks from climate change in semiarid regions of developing countries, it’s important to obtain a greater understanding of the perception of risk and adaptation of human communities located in the Brazilian semiarid region, since according to the IPCC, rural communities, and locations in developing countries will be among the main affected by extreme climate events soon [1].

Thus, this work aims to understand which factors shape the perception and adaptation of a community of rural farmers in the semiarid region of Pernambuco in the face of health risks caused by climate change. For this, we built four questions, with their respective hypotheses and predictions.

One of the questions in this study is: how socioeconomic factors influence the perception of health risks during extreme climate events? The hypothesis one (H1) is that income, sex, age, family size, education and access to health services influence the amount of perceived risks in periods of drought or extremes rainfall. The prediction is that all these factors will have a positive impact on the amount of perceived risk in both extreme climate events.

Another issue raised is: how socioeconomic factors affect the adoption of adaptive responses to mitigate health risks during extreme weather events? The hypothesis two (H2) is that income, sex, age, family size, education and access to health services influence the amount of adaptive responses used during periods of drought or excessive rainfall. The prediction is that all these factors will have a positive impact on the number of adaptive responses used in both events.

In addition, the study seeks to understand: how the amount of perceived risk affects the amount of adaptive responses used? Hypothesis three (H3) is that the amount of adaptive responses^1^ used is directly influenced by the amount of perceived risk in both extreme climate events. The prediction is that the number of adaptive responses used will positively influence the number of perceived risks.

Finally, the study intends to investigate: what’s the influence of extreme weather events on the amount of perceived risks and the amount of adaptive responses used? The hypothesis four (H4) is that drought, the most frequent climatic event, has a greater impact on the amount of perceived risks and adaptive responses used. The prediction is that the amount of perceived risks and the amount of adaptive responses used will be greater during periods of drought compared to periods of extremes rainfall.

## Methods

### Study area

**Fig. 1:**
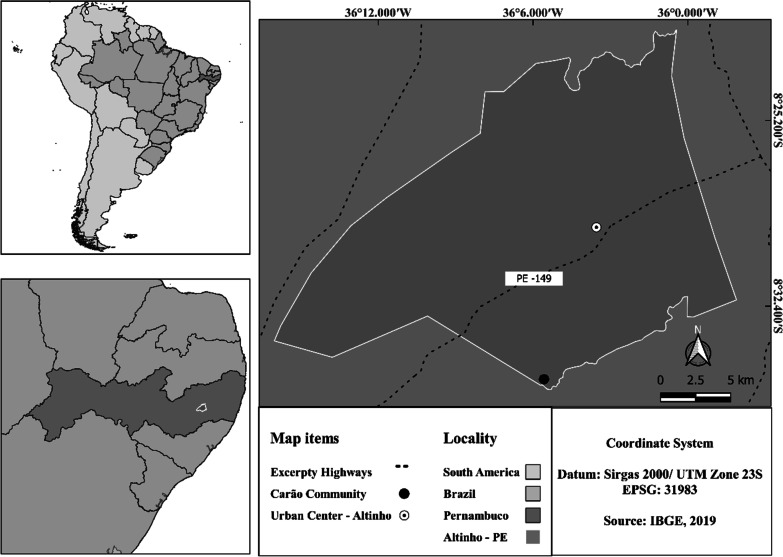
Location of the Carão community in the state of Pernambuco, Brazil, South America

The study was carried out in the community of Carão (Fig. 1) (08° 35′ 13.5″ S and 36° 05′ 34.6″ W), which is in the rural area of Altinho, Pernambuco—Northeastern Brazil. The community is approximately 16 km from the nearest urban center in the central region of the municipality of Altinho [28]. According to the Köppen-Geiger climate classification, the climate in the region is of the “BSh” type; therefore, the community is in one of the semiarid regions of the planet. The wet season occurs between June and July, with rainfall levels approaching 746 mm [29]. In Fig. 1, it’s possible to observe a map indicating the location of the Carão community in Pernambuco, Brazil.

According to Martins et al. [30], the region where the community is located is identified as a risk area for drought events. The short rainy season, added to the great risks of drought, imposes intense drought regimes on the community area, which have become more recurrent in NE Brazil [30]. The municipality of Altinho is one of the constituents of the polygon of droughts, a sociogeographical region delimited by the Brazilian government, which is characterized by the aridity of the environment, with prolonged periods of drought and low socioeconomic development [30].

The characteristics of the semiarid region confer a greater scarcity in the water regime, and therefore, economic problems such as difficulties in agricultural practice, forcing populations to use forest resources as sources of energy, food, therapeutic use and raw material for building homes [31–36].

### Socioeconomic characterization of the community

**Fig. 2:**
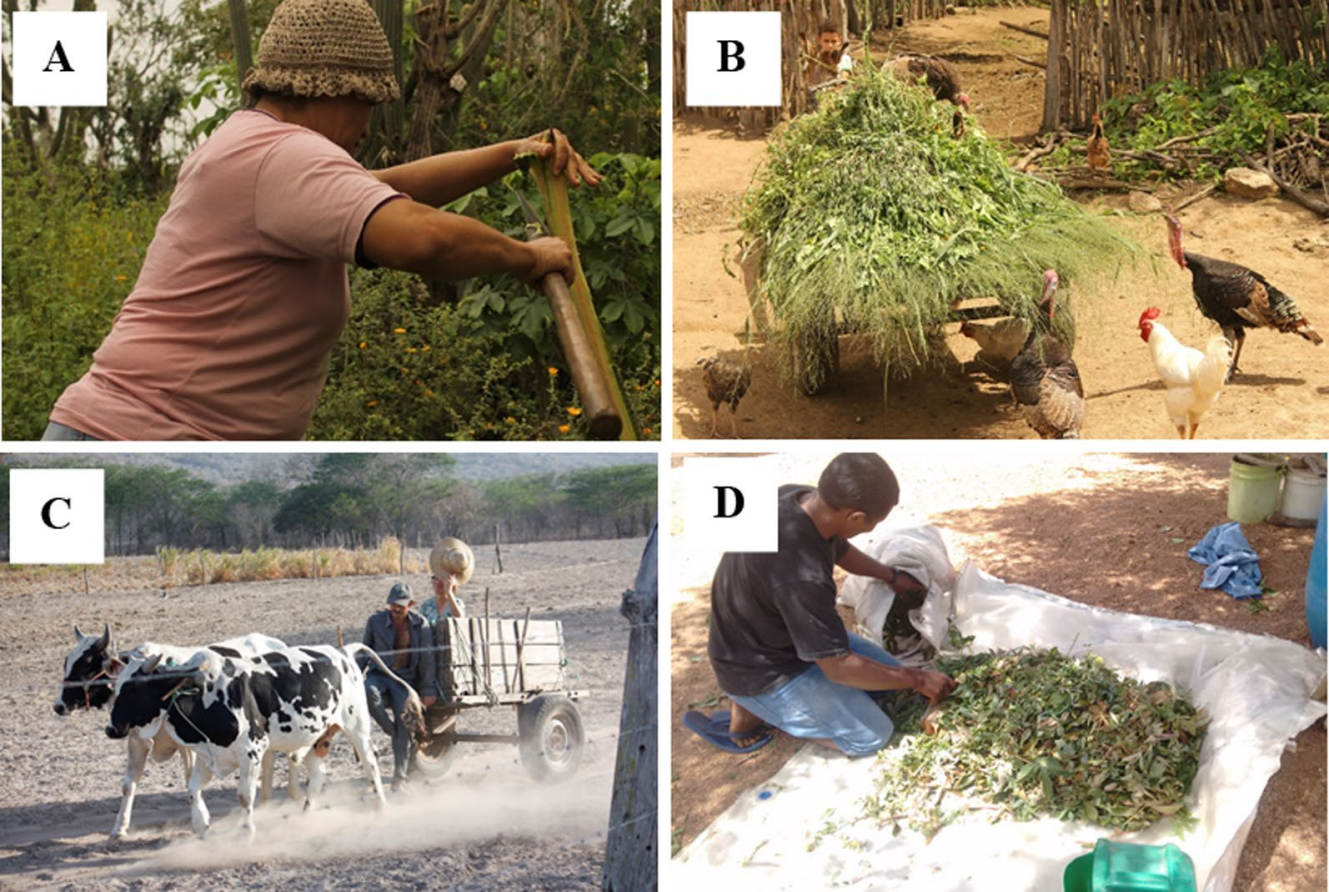
**A** Extraction of feed plants for livestock. **B** Transportation of plants for poultry feed production. **C** Use of cattle for fetching water from reservoirs. **D** Preparation and drying of leaves for make tea.Images by Flávia Santoro/Image bank of the Laboratory of Ecology and Evolution of Social-ecological Systems

The Carão community has a public health center that is responsible for serving the population of Carão and other nearby communities. According to the information obtained from the health center, the community has 108 residents over the age of 18. It was also revealed that agriculture is the main occupation within the community. Despite agriculture being the main occupation in the community for approximately 10 years [37], some of the residents, in general, the younger ones, have been looking for other sources of income, mainly in the nearest urban centers (Altinho and Caruaru). These individuals are characterized by having a double home, as they work in urban centers and therefore remain there during working days, returning to the community on weekends. In Fig. 2, it is possible to observe typical labor activities within the Carão community.

To conduct our research, we sought volunteers living in Carão of both biological sexes who were over 18 years old and willing to participate in the study. During the period in the community, we conducted 5 visits over the course of 1 year, and visited all occupied residences, in which we interviewed 49 people, this being the total number of our sample, which corresponds to 45.37% of the population of Carão. Among the 49 volunteers, 28 were biologically female, corresponding to 57% of the sample, and 21 were biologically male, corresponding to 43% of the sample.

Approximately 75% of the volunteers were literate and had schooling up to elementary school, 12% had completed high school, and 12% were not literate. Families have an average of three people per residence, with the largest family consisting of five people and the smallest, one person. The average monthly income of volunteers is U$312.23,^2^ ranging from US$35.92 to US$758.29 per family. Ninety-three percent of the volunteers reported having access to public health services; on the other hand, 9% claimed not to have access to such services. Data on the sociodemographic profile of the Carão community can be seen in Table 1.

**Table 1:** Sociodemographic profile of the Carão community, Altinho, Northeastern Brazil

Sociodemographic characteristics	Percentage
Residents over 18 years old	
Total 108	100%
Sample 49	45.37%
Sex-Sample	
Female 28	57%
Male 21	43%
Schooling—sample	
High School 6	12%
Elementary School 37	75%
Not literate 6	12%
Income—sample	
Less than US$ 214.29 (minimum wage)	18%
Between $214.29 and $428.58 31	64%
Above $428.58 9	18%
Access to health services—sample	
Yes 43	93%
No 3	9%
Family size—sample	
Between 1 and 3 people 34	70%
Between 3 and 5 people 15	30%

### Ethical and legal aspects

This work is linked to the project *Perception of environmental risks and adaptive capacity to climate change in socioecological systems in the Brazilian semiarid region*, led by Henrique Fernandes Magalhães, which was submitted to the Research Ethics Council of the University of Pernambuco (CEP/UPE) and the National Research Ethics Commission (CONEP), being approved under number 4.498.835. Volunteers selected to participate in the study signed the Free and Informed Consent Form, in accordance with Resolution no. 466/2012 of the National Health Council, which authorizes the collection, use and publication of available data.

### Data collection

The Laboratory of Ecology and Evolution of Social-ecological Systems (LEA) has been conducting research in the community for approximately 13 years. Therefore, continuous communication between the group's researchers and the Carão community made the first contact about the present work feasible. Three visits were made to the community in July 2019, December 2019, and February 2020. On the first visit, we held a meeting with community residents at the local association, focusing on explaining the research objectives. After the meeting, the community agreed to be part of the study.

The volunteers were submitted to semistructured interviews [38] in which, in the first stage of the interview, we sought information about socioeconomic profiles. Initially, the volunteers were asked about age, sex, education, access to public health services, family size and family monthly income. Later, in a second stage of the interview, we asked the volunteers to list the years with prolonged droughts in the region, as well as the years with intense rain. In a third step, we asked them to also list the health risks that were more frequent in years when there were prolonged droughts and the most frequent health risks in years when there was heavy rainfall. In a fourth stage of the interview, we asked the volunteers to list the types of behavior, use of herbal medicine/medication, or any type of responses they used to face each health risk mentioned above.

To achieve greater methodological precision in the study, the questions were asked to obtain qualitative data, as we sought to know what the perceived risk was, what was the response used, and in which years did the greatest droughts and rains occur in the region. The questions were asked in this way to encourage the volunteers to provide us with information as accurately as possible, since if we asked for the quantity of each item, it would be difficult for them to tell us exactly how many risks they perceive, how many response they use and how many times droughts have occurred or rains.

### Data analysis

For the statistical analysis of the data, we transformed the cited risks and the adaptive responses used into quantitative variables. Therefore, each risk cited and each response used was converted into discrete quantitative variable. The categorization of qualitative data into quantitative data was carried out as follows: we asked the volunteer to cite health risks during periods of drought and extremes rainfall. Ultimately, we counted how many risks the volunteer had mentioned. For example, if the volunteer cited the risks dengue and flu and the response to risk its tea and drug, this volunteer cited two risks and two adaptative responses.

The socioeconomic data collected were used as predictor variables in hypotheses 1 and 2. The variables are age, sex, education, access to public health services, family size and monthly family income. The age variable was categorized into three different levels: youth, adults and elderly. Youth included volunteers aged 18 to 25, adults aged 26 to 60, and seniors aged 61 to 100. The schooling variable was categorized into three distinct levels: complete high school, elementary school and illiterate. The variable “access to health services” is dichotomous; therefore, its answers are standardized as “yes” for volunteers who claim to have access and “no” for volunteers who claim not to have access to health services (Table 2).

**Table 2:** Set of socioeconomic variables used in the study, how they were categorized, their composition and type (categorical or continuous)

Variable	Type	Composition
Sex	Categorical	Female and male
Age	Categorical	Young people, adults and seniors
Income	Quantitative	$35.92–$758.29
Access to health services	Categorical	Yes or no
Family size	Quantitative	0–5 residents
Education	Categorical	Nonliterate, elementary school and high school

In the analysis of the first hypothesis of the study, which assesses whether socioeconomic variables influence the perception of risks, we built two generalized linear models, one for each climate extreme (Table 3).

**Table 3:** Demonstration of the variables used in the test of the H1 in each generalized linear model

Model	Predictor variable	Response variable
1	Socioeconomic variables	Amount of risks cited in years with prolonged droughts
2	Socioeconomic variables	Amount of risks cited in years with heavy rainfall

In the analysis of the second hypothesis of the study, which assesses whether socioeconomic variables influence the use of adaptive responses, we also built two generalized linear models, one for each climate extreme (Table 4).

**Table 4:** Demonstration of the variables used in the test of the H2 in each generalized linear model

Model	Predictor variable	Response variable
1	Socioeconomic variables	Amount of adaptive responses used in years with prolonged droughts
2	Socioeconomic variables	Amount of adaptive responses used in years with heavy rainfall

For the third hypothesis of the study, which assesses whether the amount of adaptive responses used is directly influenced by the amount of perceived risks in both climate extremes, two generalized linear models were built (Table 5).

**Table 5:** Demonstration of the variables used in the test of the second third in each generalized linear model

Model	Predictor variable	Response variable
1	Perceived risks in years with prolonged droughts	Amount of adaptive responses used in years with prolonged droughts
2	Perceived risks in years with heavy rains	Amount of adaptive responses used in years with heavy rainfall

In all models of the three hypotheses mentioned, we used the Poisson distribution, as the data obtained do not follow a Gaussian distribution, in addition to being a discrete quantitative variable. After the analyses, the four models were submitted to mathematical refinement using the stepwise method. We used the stepwise refinement method to develop models with the most explanatory variables.

For the fourth hypothesis, we seek to understand whether the most frequent climatic extreme influences the perception of risk and the use of adaptive responses. We used the nonparametric Mann‒Whitney test, with the objective of comparing the amount of risks perceived in years with prolonged droughts with the amount of risks perceived in years of rain. The same test was used to compare the amount of adaptive response used in years with prolonged droughts with the amount of adaptive responses used in years of intense rain.

In addition to the analysis of the hypotheses, we also prepared descriptive analyses of the numerical independent variables (Table 6). All statistical analyses used in this study were conducted in the R development environment, version 4.0.3 [39].

**Table 6:** Descriptive statistics of numeric independent variables

Variable	Minimum	Maximum	Average	Median	Standard deviation	Variance
Income^a^	180R$	3500R$	1564R$	1075R$	998.2507	996,504.5
Age	23 years	80 years	55 years	58 years old	15.2014	231.0825
family size	1 person	5 people	3 people	3 people	1.221965	1.493197

^a^The values used to carry out the statistical analyzes refer to the Brazilian currency

## Results

In our best explanatory model for the influence of socioeconomic variables on risk perception in times of drought (Akaike Information Criterion—AIC = 175.26), only income had a significant effect (*p* < 0.03; *z* = 2.238)—(Table 7); therefore, our H1 was not supported. In the best explanatory model related to periods of prolonged rainfall (AIC = 148.73), in turn, only age in the elderly category explained the perception of risks to human health (*p* < 0.0001; *z* = − 3.302)—(Table 8). As, in both models, only one socioeconomic variable had a significant effect, our hypothesis was not supported, since our H1 expected that the set of socioeconomic variables influenced risk perception and.

**Table 7:** Generalized Linear Model between socioeconomic variables and perceived risks of drought season

Variable	Estimate	Standard error	*z* value	*p* value
Male	− 0.3509741	0.2267279	− 1.548	0.1216
Income	0.0002398	0.0001072	2.238	0.0252

**Table 8:** Generalized linear model between socioeconomic variables and perceived risks in rainy season

Variable	Estimate	Standard error	*z* value	*p* value
Age—Elderly	− 0.8781959	0.2659865	− 3.302	0.000961
Age—Young	− 0.3544313	0.6016500	− 0.589	0.555795
Income	0.0002359	0.0001336	1.765	0.077590
Family size	− 0.2165127	0.1217269	− 1.779	0.075293

As in the previous hypothesis, we expected a joint action of the predictive variables on the number of adaptive responses used. Our hypothesis was not supported since none of the variables significantly explained the phenomenon, and the most explanatory model presented an AIC value = 163.93 (Table 9).

**Table 9:** More explanatory generalized linear model between the socioeconomic variables and the adaptive responses used in years of drought

Variable	Estimate	Standard error	*z* value	*p* value
Male	− 0.3906951	0.2353150	− 1.660	0.0969
Income	0.0001851	0.0001132	1.635	0.1019
Access to health-Yes	1.3066897	1.0189995	1.282	0.1997

In our second model, age in the elderly category (*p* < 0.0001; *z* = − 3589), male sex (*p* < 0.05; *z* = − 2.109) and family size (*p* < 0.0001; *z* = − 3.110) explained adaptive responses in periods of intense rainfall (Table 10). The most explanatory model presented AIC = 161.67.

**Table 10:** Generalized linear model between socioeconomic variables and adaptive responses used in rainy season

Variable	Estimate	Standard error	*z* value	*p* value
Age—Elderly	− 0.8819313	0.2457079	− 3,589	0.000332
Young age	− 0.7512808	0.7304233	− 1.029	0.303689
Male	− 0.4674316	0.2216572	− 2.109	0.034961
Income	0.0002537	0.0001331	1906	0.056670
Family size	0.3609938	0.1160790	− 3,110	0.001872

Our hypothesis about the relationship between perceived risks and the adaptive s used was confirmed and is independent of the climatic extreme in question (Table 11). The model obtained for dry seasons presented *p* < 0.0001 and *z* = 6.422 with AIC = 130.08. The model for rainy seasons presented values of *p* < 0.0001; *z* = 5.012 with AIC = 148.36.

**Table 11:** Generalized linear model between the perceived risks in times of drought and the adaptive responses used in the same season/perceived risks in times of rain and the same season

Variable	Estimate	Standard error	*z* value	*p* value
Perceived risks in years of drought	0.39702	0.06183	6422	0.0000000000135
Perceived risks in rainy years	0.45491	0.09077	5012	0.0000000539

We found no significant difference between the amount of perceived risks and the amount of adaptive response in both climatic extremes, even though prolonged droughts were more frequent than periods of extremes rainfall (Table 12). Therefore, our hypothesis was not supported. The value of the Mann‒Whitney test for comparing the amount of perceived risks between dry and wet years was *p* = 0.8121 and = 1167.5. The value of the Mann‒Whitney test for comparing the number of adaptive responses used in dry years and rainy years was *p* = 05356 and *w* = 1115.

**Table 12:** Descriptive statistics of dependent variables

Variable	Minimum	Maximum	Average	Median	Standard deviation	Variance
Risks in years of drought	0	6	1.673	1	1.625164	2.641156
Risks in rainy years	0	4	1.592	1	1.188808	1.413265
Responses in drought years	0	5	1.592	1	1.442197	2.079932
Responses in rainy years	1	7	1.816	Two	1.576831	2.486395

## Discussion

The scientific literature that studies the effects that affect the perception of risk has used a set of socioeconomic variables as predictors for the perception of health risks; however, not all of them were significant. In Chan et al. [40] for example, the authors used variables such as sex, education, age, family income and occupation to try to understand which profiles perceived with greater and less severity the risks arising from the COVID-19 pandemic in citizens of Hong Kong. As a result, income and age were significant variables. In this study, people with the lowest incomes perceived that they were more likely to be financially affected by the pandemic. In turn, people with more advanced ages (elderly people over 65 years old) perceived themselves to be less prone to social, financial, and health risks arising from the pandemic.

Income has been widely used in studies that seek to highlight the influences of this factor on risk perception [41, 42]. In general, families with lower incomes are more susceptible to the local impacts of climate change [43, 44], as well as its adjacent events, such as diseases caused by droughts and floods [45, 46]. Therefore, they tend to perceive more risks or risks with greater severity [43, 44].

However, our results show that there is a positive linear relationship between income and the amount of health risks perceived in times of drought; therefore, people with higher incomes tend to perceive more risks in this situation, not following the trends found in the literature and cited above. We believe that, in our scenario, income was a significant factor in risk perception because according to Hovick et al. [47]. Peoples with higher incomes have greater access to information about health care; in contrast, families with lower incomes are more likely to seek less information [48]. This study, conducted by the National Cancer Institute, aimed to investigate the characteristics of individuals seeking health information, based on a sample of 6133 cancer patients. The findings revealed that individuals with lower income and education levels exhibited lower levels of information-seeking behavior. Additionally, these individuals demonstrated lower scores in health preventive behaviors [48]. This study sheds light on the association between socioeconomic factors and health-related behaviors, emphasizing the need for targeted interventions to ensure equitable access to information and promote preventive measures among all segments of the population.

Access to information is not necessarily linked to the schooling variable, since, in our study, this variable only considers the level of schooling obtained through educational institutions, and information about droughts and their evils can be learned through other means, such as television and the internet. In Rauf et al. [49] for example, the authors argue that information obtained from sources such as family and friends, newspapers, television and the internet plays a fundamental role in the perception of health risks in a context of waves of heat.

Additionally, only the age variable affected individuals' perception of risk in rainy seasons. Our results demonstrate that there is a negative relationship between age in the elderly category and the perception of risk since with increasing age, the amount of perceived risk decreases. The literature shows that older people in general tend to perceive and worry more about health risks than younger people; [50] however, our findings differ from what has already been found in the literature.

Botzen et al. [51] and Akerlof et al. [52] comment that although advanced age is a factor that confers greater vulnerability, this is not necessarily associated with an increase in risk perception. According to Leiserowitz [53] vulnerability can be one of the factors that modulate the perception of risk. Therefore, we believe that not being aware of their own vulnerability may lead the elderly to perceive fewer health risks in rainy seasons. This finding agrees with [54] who found evidence in which the elderly did not recognize themselves as more vulnerable people and therefore perceived less risks.

The scientific literature has an extensive history of how socioeconomic variables influence the use of adaptive responses [27, 55, 56]. Our findings demonstrate that this is not a determining factor for the adaptation of individuals in the face of health risks caused by drought. We believe that the fact that drought is very frequent in the region [57] made the adaptive capacity of individuals more homogeneous and therefore not influenced by the socioeconomic variables used in the study. The fact that no variable is significantly important in the adaptation processes during drought indicates that the combined use of these variables may not be ideal for studies in the Brazilian semiarid region. Therefore, we suggest that future studies select different variables, with hypotheses based on each variable and not on a joint action. For example, in the study conducted by Sena et al. [45], the authors argue that studying sex differences in adaptation to drought can help us understand adaptive patterns in regions vulnerable to this climate extreme.

On the other hand, our findings indicate that the socioeconomic factors age (in the elderly category), sex (in the male category), and family size influence the amount of adaptive responses used in rainy seasons. Elderly people (people aged 65 years or older) are often cited in the literature as one of the most vulnerable groups to climate change and its consequences [54, 58]. Our results indicate that there is a negative linear relationship between the elderly category and the use of adaptive responses in rainy seasons; therefore, the elderly in the community of Carão use fewer responses during these periods.

Gamble et al. [59] cite some factors that may determine the use of adaptive responses by elderly individuals. Among these, physiological limitations and mobility impairments inherent to elderly people stand out. The authors argue that the decrease in motor coordination, as well as the decrease in cognitive function, limits the adaptive behavior in these individuals. We believe that these factors may limit the elderly in the community in the use of adaptive responses.

The results also indicate that male individuals use fewer adaptive responses in coping with health risks. These results are in line with those Rauf et al. [49] that pointed to sex as a major determinant in the use of adaptives responses. In this study, the authors emphasize a notable gender difference in adaptive behaviors within the context of family health care, with men exhibiting fewer adaptive behaviors compared to women. This discrepancy can be attributed to their limited involvement in social roles associated with the care of children and other family members, leading to a decreased inclination towards adaptive responses. Family size also plays an influential role, according to the results. The positive linear relationship indicates that people with more family members tend to use more adaptive responses. Our findings are consistent with those of Williams et al. [60]. The study's results indicate that individuals with larger families tend to demonstrate concerns for a greater number of people, suggesting a higher likelihood of employing a greater number of adaptive responses. This suggests that individuals with larger families are more likely to utilize a wider range of adaptive strategies, potentially due to their increased responsibilities and considerations for multiple family members.

The amount of risks perceived in dry seasons and in rainy seasons also function as determining factors in the amount of adaptive responses used. The results of our third hypothesis demonstrate the existence of a linear relationship between the perceived risks and the adaptive responses used, and this happens both in dry and rainy seasons, which reveals to us what the strong alignment of perceptions with responses is context-independent. In addition, our fourth hypothesis found that there are no differences between the amount of perceived risks in dry and rainy seasons, and there are no differences in the amount of adaptive responses used in both periods. Our findings find interesting support in the literature, as in Budhathoki et al. [61] for example, which found that risk perception influences the adaptive responses of farmers in Nepal. Another interesting example, already in the context of adapting to risks that affect health, comes from the work conducted by Ban et al. [62]. In this study, the authors found that the perception of the risk of heatwaves is a good predictor of the adoption of adaptive responses.

## Conclusion

Faced with the challenges posed by global climate change, it is increasingly important to understand how different populations deal with the health risks arising from extreme climate events. In this sense, this study investigated the risk perception and adaptive responses of small farmers residing in semiarid regions of NE Brazil. The results showed that the amount of adaptive responses used by rural farmers is directly influenced by the amount of perceived risks, regardless of the type of climate extreme faced. In addition, the study emphasizes the importance of considering socioeconomic factors in the analysis of risk perception and the adoption of adaptive responses in extreme weather events. Our findings contribute to understanding the complexity involved in risk perception and the adoption of adaptive responses in extreme weather events. In addition, the results highlight the importance of considering risk perception as a central element for planning adaptation measures. This approach can contribute to promoting more effective adaptation measures and reducing the impacts of climate change on the health of the most vulnerable populations.

Finally, it is important to acknowledge certain limitations of this study. Firstly, the sample size of small farmers in the semiarid regions may not be representative of the entire population, thus limiting the generalizability of the findings. Additionally, the study focused solely on the perspective of small farmers and did not incorporate the viewpoints of other stakeholders.

Therefore, further research involving a more diverse range of participants and perspectives is warranted. Additionally, it should be noted that the study relied on self-reported data, which may be susceptible to recall bias or social desirability bias. The subjective nature of self-perception regarding risks and adaptive responses may introduce variations and interpretations among individuals.

Future studies could consider employing objective measurements or incorporating multiple sources of data to enhance the validity and reliability of the findings. In conclusion, while this study provides valuable insights into the risk perception and adaptive responses of small farmers in semiarid regions, it is crucial to recognize the cited limitations. Addressing these limitations and conducting further research will contribute to a more comprehensive understanding of the complexities surrounding risk perception and the adoption of adaptive responses in extreme weather events. Moreover, it will support the development of tailored and effective adaptation policies that address the specific needs and vulnerabilities of local communities.

## Data Availability

The data that support our findings of this study are available from the corresponding author upon request.

## Footnotes

1 In this study, we are considering adaptive responses and adaptive strategies as the same phenomenon.

2 Amount of R$1564 converted to US dollar (USD) at the exchange rate on 04/11/2022.
